# Investigation of COVID-19 Misinformation in Arabic on Twitter: Content Analysis

**DOI:** 10.2196/37007

**Published:** 2022-07-26

**Authors:** Ahmed Al-Rawi, Abdelrahman Fakida, Kelly Grounds

**Affiliations:** 1 School of Communication Simon Fraser University Burnaby, BC Canada

**Keywords:** COVID-19, Arab world, Twitter, misinformation, vaccination, infodemiology, vaccine hesitancy, infoveillance, health information, social media, social media content, content analysis, Twitter analysis

## Abstract

**Background:**

The COVID-19 pandemic has been occurring concurrently with an infodemic of misinformation about the virus. Spreading primarily on social media, there has been a significant academic effort to understand the English side of this infodemic. However, much less attention has been paid to the Arabic side.

**Objective:**

There is an urgent need to examine the scale of Arabic COVID-19 disinformation. This study empirically examines how Arabic speakers use specific hashtags on Twitter to express antivaccine and antipandemic views to uncover trends in their social media usage. By exploring this topic, we aim to fill a gap in the literature that can help understand conspiracies in Arabic around COVID-19.

**Methods:**

This study used content analysis to understand how 13 popular Arabic hashtags were used in antivaccine communities. We used Twitter Academic API v2 to search for the hashtags from the beginning of August 1, 2006, until October 10, 2021. After downloading a large data set from Twitter, we identified major categories or topics in the sample data set using emergent coding. Emergent coding was chosen because of its ability to inductively identify the themes that repeatedly emerged from the data set. Then, after revising the coding scheme, we coded the rest of the tweets and examined the results. In the second attempt and with a modified codebook, an acceptable intercoder agreement was reached (Krippendorff α≥.774).

**Results:**

In total, we found 476,048 tweets, mostly posted in 2021. First, the topic of infringing on civil liberties (n=483, 41.1%) covers ways that governments have allegedly infringed on civil liberties during the pandemic and unfair restrictions that have been imposed on unvaccinated individuals. Users here focus on topics concerning their civil liberties and freedoms, claiming that governments violated such rights following the pandemic. Notably, users denounce government efforts to force them to take any of the COVID-19 vaccines for different reasons. This was followed by vaccine-related conspiracies (n=476, 40.5%), including a Deep State dictating pandemic policies, mistrusting vaccine efficacy, and discussing unproven treatments. Although users tweeted about a range of different conspiracy theories, mistrusting the vaccine’s efficacy, false or exaggerated claims about vaccine risks and vaccine-related diseases, and governments and pharmaceutical companies profiting from vaccines and intentionally risking the general public health appeared the most. Finally, calls for action (n=149, 12.6%) encourage individuals to participate in civil demonstrations. These calls range from protesting to encouraging other users to take action about the vaccine mandate. For each of these categories, we also attempted to trace the logic behind the different categories by exploring different types of conspiracy theories for each category.

**Conclusions:**

Based on our findings, we were able to identify 3 prominent topics that were prevalent amongst Arabic speakers on Twitter. These categories focused on violations of civil liberties by governments, conspiracy theories about the vaccines, and calls for action. Our findings also highlight the need for more research to better understand the impact of COVID-19 disinformation on the Arab world.

## Introduction

### Background

The COVID-19 pandemic has occurred in tandem with what is being called an *infodemic,* referring to the large amounts of false and misleading content about the virus being disseminated primarily over social media platforms [1]. Although English COVID-19 disinformation and misinformation research and data sets have received extensive attention [2], content shared in other languages, specifically Arabic, has been neglected [3,4].

Misinformation is not a new phenomenon in the Arab region [5]. Some scholars have focused on the reach and effects of Arabic misinformation during the pandemic [2], while others have included Arabic misinformation as a secondary focus [6]. Nevertheless, there is an urgent need for an examination of the scale of Arabic COVID-19 disinformation. Arabic, for example, is currently Facebook’s third-most common language [7] and a language spoken by more than 400 million people [8] in countries with significant social media presence [9]. Hence, this study attempts to fill a gap in the literature by examining social media data retrieved from Twitter to better understand the main online discussions that revolve around COVID-19 misinformation.

There seems to be an abundance of misinformation in non-English discourses that social media companies largely ignore, for they do not often react to viral Arabic COVID-19 misinformation with the same urgency they have for English misinformation. Facebook took some measures to address English COVID-19 misinformation, but the platform applied limited measures to other languages [10]. For example, Facebook fact-checks viral information in Western countries through fact-checking partners, yet such debunking is not applied to the same information when translated to Arabic [10]. Various spoken Arabic dialects pose a challenge to Facebook’s algorithms and human moderators, given each dialect’s unique vocabulary and historical and cultural contexts [7]. Transcribing Arabic varies between native speakers from different countries, and as Facebook continues to rely on artificial intelligence to moderate content, the platform will continue to misinterpret Arabic posts [7].

There is a pressing need to thoroughly understand and examine the social media platforms where Arabic COVID-19 disinformation spreads. Current studies focus on Facebook pages and groups given their popularity, especially in some Arab countries [11,12]. However, Facebook is not the only platform where Arabic COVID-19 misinformation is spread [8]. Content on Facebook has found its way to Twitter accounts and YouTube channels [10], where other scholars have then analyzed Arabic COVID-19 tweets and hashtags [2,4,6]. Reviewing the use of religious misinformation during the pandemic, it was found that misinformation in YouTube videos can receive millions of views [13]. These same videos were found circulating on messaging platforms, such as WhatsApp. As 1 of the world’s most popular messaging platforms and a significant source of COVID-19 misinformation [14], WhatsApp is suggested to be a vital source of rumors, with usage surpassing Twitter in Arab countries, such as Saudi Arabia [9]. Taken together, these studies provide an overview of the online ecosystem that allows Arabic COVID-19 disinformation to spread. Our study attempts to contribute to the existing knowledge by answering the following research question: what are the major topics that are discussed around COVID-19 misinformation in Arabic on Twitter?

### Literature Review

Although various disinformation and misinformation narratives in Arabic spread on social media range from “full-throated conspiracy theories to unscientific health advice” [9], our review of the literature found that there is a focus on 3 categories of disinformation. These 3 categories are COVID-19 disinformation, government-targeted disinformation, and religious disinformation. COVID-19 disinformation tends to focus on the symptoms of the virus, its vaccine, alternative remedies, and theories about its origins. Government-targeted disinformation looks at content that addresses specific governments and the health measures that they have taken. Finally, religious disinformation looks at misleading advice given by religious leaders and misinterpretations of religious texts during the pandemic and its potential to cause fear and confusion. In addition to these categories, we also found a smaller body of literature that discussed the origins of the Arabic disinformation, focusing on the regions that it emerges from and the governments that sponsor it. Each of these categories will be discussed later.

### COVID-19 Disinformation

Disinformation about the COVID-19 virus and its vaccination has received ample attention from social media users. The disinformation includes false information about the virus and its treatment, the promotion of inaccurate or false claims about home remedies as alternatives to existing treatments, and conspiracy theories about the virus’s origins [12]. This disinformation has also been noticeably popular in Iran, with users engaging with it consistently. One popular false remedy that appeared to Iranian users touted alcohol as a cure for COVID-19. This false remedy resulted in at least 27 people dying of alcohol poisoning [15]. This example shows the potential impact of misinformation that caused real-life harm.

In Arab countries, misinformation about topics such as the virus’s origins or government-related conspiracies about the vaccine have begun to widely appear on social media as legitimate sources of information. A paper exploring COVID-19 misinformation in Jordan found that different Arabic media outlets amplified conspiracy theories about the pandemic and the most common conspiracy theories amplified tended to focus on the virus’s origins [16]. The majority of these theories stated that the virus was either created in a lab as part of a biologic warfare campaign or was caused by the 5G network [17]. In the paper’s sequel, the authors found that these same narratives had persisted, with the theories that the virus came out of a lab remaining the most prominent [17]. The study also showed a growing connection between conspiracy theories and disinformation about the treatment and vaccine. Most of these conspiracy theories claim that the long-term effects of both treatments are unknown and could potentially be dangerous [16]. Like social media content that focuses on the virus and its treatments, conspiracy theory content also poses a significant risk to public health efforts by continuing to propagate antivaccine theories that could lower the overall rates.

### Government-Targeted Disinformation

Government-targeted disinformation includes speculation that they are co-opting the pandemic to impose new restrictions on individuals or a manipulation of official government statements to fit a narrative. It is important to note that government-targeted disinformation is different from posts that discuss the Deep State and other conspiracy theories, such as the New World Order. Posts about the Deep State and other groups are based on unfounded claims and about their role in the pandemic and appear in the previous category of COVID-19 disinformation [18]. In contrast, government-targeted disinformation uses actual statements and actions taken by different governments and twists them to shape a false narrative.

There are 2 subcategories of government-targeted disinformation. The first targets the pandemic responses and policies of Arab governments, while the second focuses on non-Arabic governments and falls under the conspiracy theory category. Government-focused disinformation tries to co-opt information and statements to use against the rulers. Most of the content takes the statements of government officials out of context to put their pandemic responses in a negative light [12]. Based on additional analysis, this type of disinformation was the most prevalent type on Egyptian social media, while also having the lowest engagement of all 4 content categories [12]. The observed lower levels of engagement are likely because this category of disinformation rarely attached videos or sources to its posts, meaning that users were less likely to engage with it [12].

The disinformation that utilizes manipulated pandemic-related content has been shown to have some of the highest levels of engagement. Another paper argues that these higher levels of engagement are a result of these posts imitating and co-opting sections of actual news articles to increase their perceived legitimacy [15]. This is echoed by other scholars who found that on Egyptian social media, in particular Facebook, manipulated content was 1 of the most prominent types of disinformation to be shared. Within this category of disinformation, posts that reconfigured existing news articles to agree with a false narrative were the most popular [12]. However, the manipulated content was not limited to existing news articles. One of the more prominent examples shared with Egyptian Facebook users was a clip from a documentary manipulated to show that an asteroid would hit the earth once the COVID-19 pandemic ended [12]. Of all 3 categories, this content has the most significant potential to create panic by using posts that appear legitimate, creating a sense of trust with the user.

### Religious Arabic Disinformation

The final category, religious disinformation, tends to take the form of misleading advice, and misinterpretations of religious scriptures are vital to consider, given Islam’s role in the region culturally, socially, and politically [13]. Analyzing the growth of religious misinformation during the COVID-19 pandemic in the Arab region, 1 paper highlights the ability of actors in the region to promote their misinformation, endanger public health, and cause confusion and fear [13]. This type of misinformation includes top-down religious misinformation coming from authority figures. In Iran, clerics have been spreading top-down misinformation, while there has also been bottom-up misinformation disseminated by content creators aiming to attract followers and subscribers [13]. These actors promoted fake remedies through religious misinformation and took advantage of the pandemic-induced uncertainty [13]. However, regardless of the original source of disinformation, it still poses a significant risk to public health.

### Sources of Arabic Disinformation

The majority of the literature on the topic of COVID-19 conspiracies focuses on the different types of disinformation with which users interact. However, to date, there has been less of an emphasis on the origins of the content. The literature that does explore this tends to focus on content that emerges from state-sponsored campaigns with political motives [11]. The purpose of these campaigns is to shift the focus off a state's poor response at the domestic and international levels. Across the Arab world, such campaigns include religious elements and attempts to weaponize information to blame rivals [3]. Analyzing Arabic COVID-19 Facebook posts, 1 study noted that the content generated in these campaigns targets regional governments using digital marketing firms that leverage “COVID-19 to push geopolitically aligned narratives” [11]. The literature also suggests that there have been coordinated Arabic COVID-19 disinformation operations by Iran and Saudi Arabia and, to a lesser extent, narratives from Egypt and the United Arab Emirates [10].

Looking at Iran, there has been an effort to design and sponsor COVID-19 disinformation campaigns to deflect from their own government's pandemic response, while blaming 1 of their primary adversaries. One paper found that Tehran used 2 narratives to try and undermine the reputation of the United States [19]. The first narrative accused the United States of using sanctions to undermine Tehran's public health response to the virus. The second also targeted the United States and falsely accused them of creating the COVID-19 virus as part of their plans for biological warfare [19]. This was echoed in another study, having found similar narratives blaming the United States for creating the virus and accusing them of developing the virus to attack Shiites and Iran [3]. Both studies also found that Tehran used state-controlled media as its primary method of spreading disinformation, with a lesser degree of social media utilization [3,19].

The study also explored the Saudi COVID-19 disinformation campaigns and found that they also used narratives to attack and undermine their adversaries [3]. It is important to note that prior to the pandemic, different governments in the Arab Gulf had used fake news campaigns to attack opponents following a Saudi Arabia–led blockade of Qatar [20]. Since then, fake news battles in the Arab Gulf have taken a more public angle than other countries in the region [21]. Unlike Iran, the Saudis focused on Qatar but did not create their own disinformation. Instead, the Saudi regime took disinformation created by individuals and amplified it across multiple platforms, with the most appearing on Twitter [3]. Among the amplified narratives, there are 2 that appear to be spreading on a much larger scale. The first narrative stated that Qatar had known about COVID-19 since 2015, and the second was that Qatar was deliberately spreading the virus to damage Saudi's plans to diversify its economy [3]. The Saudis also relied heavily on social media platforms to spread their narratives, unlike Iran, which used its state-controlled media as its primary method. Despite the differences in dissemination, both regimes used their disinformation campaigns to smear their adversaries with narratives accusing them of having inside knowledge of the virus and using that to target and cripple the regimes.

The research conducted in a separate study addresses a less discussed source of Arabic COVID-19 misinformation [10]. The author identified 18 Facebook pages and 10 Facebook groups with a collective following of more than 2.4 million users that share COVID-19 content in Arabic. It found that “Arabic-language conspiracy hubs are masquerading as independent institutions, think tanks and research initiatives and are manipulating COVID-19 data, conducting their own research.” [10]. These sources, primarily located in Egypt, spread pandemic conspiracy narratives focusing on the apocalypse and antisemitism. The author of the study points out how the analyzed pages boost COVID-19 misinformation coming from Western countries by translating content and adding Arabic subtitles or voiceovers [10]. Another study also analyzed tweets using the viral conspiracy theory hashtag #FilmYourHospital, which encouraged people to take pictures of empty hospitals to show that the COVID-19 pandemic is a scam [6]. Using social network analysis techniques, the scholars found that “the second and third largest non-English clusters were users tweeting in Arabic” [6]. Taken together, these 2 studies highlight the high rates of Arabic disinformation, in addition to the already prevalent English content.

The consequences of the Arabic disinformation range from diminished trust in governments to potential decreases in vaccination rates and dangers to public health. Although the existing literature is limited, it does give insight into the types of content with which users might interact. Disinformation about the virus and its treatments appears to be the most dangerous of all the existing categories. As 1 author highlights, increasing vaccination hesitancy rates appear in countries engaging with such disinformation [16]. Within the literature discussing the origins of the content itself, Iran and Saudi Arabia have shown a clear preference for being involved in information wars through content that spreads conspiracy theories and targets regional governments [16]. However, a different paper argues that private actors are also motivated by political or financial reasons to spread fake news, such as state actors [21]. Moreover, religious leaders and content creators also took advantage of the panic the pandemic brought and utilized the religion of Islam for their benefit by spreading religious misinformation to gain new subscribers [13]. However, content created by Arabic conspiracy hubs needs more examination as there is a significant gap “in understanding the trends and the intersections with other conspiracy communities online” [10]. These trends and the understanding of content categories reflect the experiences of a limited number of countries and social media platforms.

## Methods

### Study Design

In this study, we used content analysis to investigate the most retweeted messages that reference the following 13 hashtags that are mostly used by Arabic-speaking antivaccine communities: #No_to_forced_vaccinations (#لا_للتطعيم_الاجباري), #Medical_freedom (#الحرية_الطبية), #No_for_vaccinating_children (#لا_لتطعيم_الأطفال), #Say_no_to_the_vaccine (#ارفضوا_التلقيح), #No_to_vaccines (#لا_للتطعيم), #Notovaccines (#لاللتطعيم), #Say_no_to_injections (#ارفضوا_التلقيح), #No_to_injections (#لا_للتلقيح), #Notoinjections (#لاللتلقيح), #Affected_by_the_injections (#متضرري_اللقاح), #Injections_complications (#مضاعفات_اللقاح), #No_to_the_new_world_order (#لا_للنظام_العالمي_الجديد), and #No_to_human_genetic_mutilation (#لا_للتعديل_الجيني_البشري). To identify these hashtags, we started our search a few days before October 10, 2021, by using 4 generic hashtags: #No_to_forced_vaccinations (#لا_للتطعيم_الاجباري), #Say_no_to_the_vaccine (#ارفضوا_التلقيح), #No_to_vaccines (#لا_للتطعيم), and #Notovaccines (#لاللتطعيم). After collecting all the tweets, we used a Python script to extract the most recurrent hashtags in the data set that helped us identify the popular 13 hashtags often used by this online Arab community.

**Figure 1:**
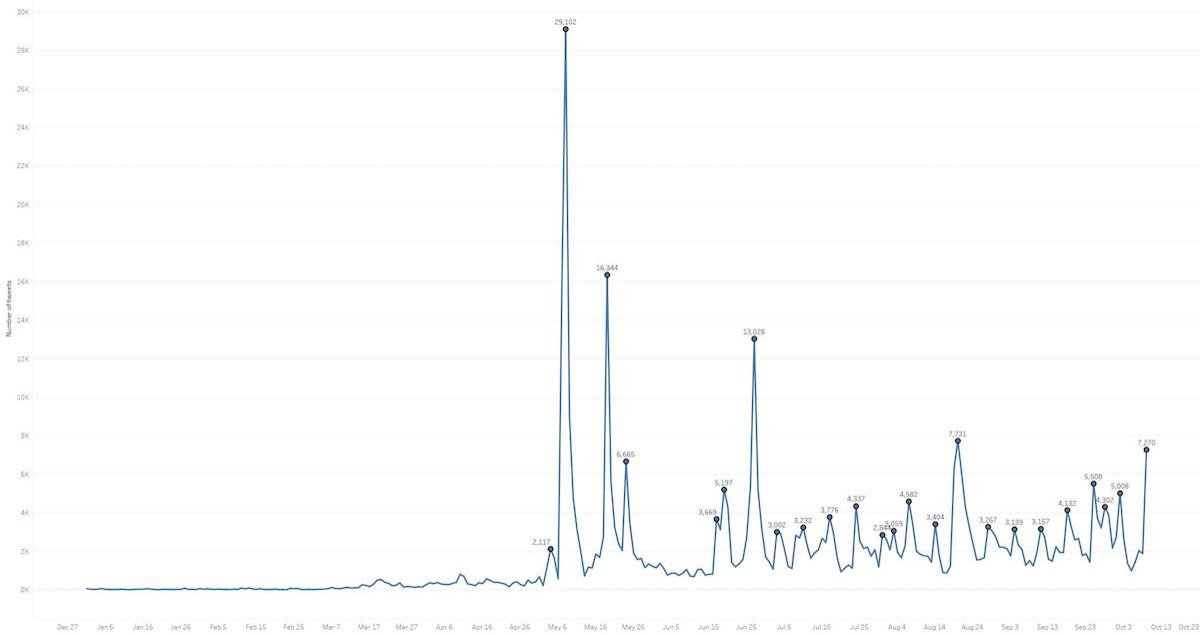
Frequency of tweets referencing 13 hashtags in 2021.

Using Twitter academic API v2 allowed us to search these hashtags from the beginning on August 1, 2006, until October 10, 2021, which is when the final data set was collected. In total, we found 476,048 tweets, mostly posted in 2021 (see Figure 1, Multimedia Appendix 1). We started from the beginning in 2006 to make sure that similar hashtags were not popular before the pandemic emerged, since this issue could distort the findings of this study. Since the data set is large, we manually analyzed the top 1000 most retweeted messages. We excluded 96 (9.6%) vague and unclear messages from the data set. By vague messages, we refer to unclear and incomprehensible tweets. For example, 1 user said in a tweet a few words like “A short story!” Another tweeted saying only the word “Kuwait.” Similar tweets that could not be analyzed were replaced with other tweets from the rest of the data set in order to have a total of 1000 retweeted messages. Our goal was to exclusively collect Arabic hashtags, but there were tweets written partly or fully in other languages, including English.

To identify major categories or topics in the sample data set, 2 coders relied on emergent coding [22] and a codebook was designed inductively that covered the range of issues found in the data set. Emergent coding was selected because of its ability to identify themes that appear repeatedly in a subset of the larger data set. These themes could then be developed into a codebook, which was used to code the entire sample in the data set. The first step of the process followed a qualitative review of 100 tweets (10% of the overall data set) by 2 coders working independently to highlight themes emerging from the data. For example, the coders noticed several tweets focusing on different vaccine conspiracy theories, which led to highlighting such themes and creating subthemes that discuss each conspiracy. Moreover, the coders noticed that a few tweets had specific calls to action that encouraged others to participate in several different activities, such as rallies or protests. We note that some of the themes that emerged were similar to the ones found in previous research that we referenced in our literature review. Based on the themes found, an initial codebook was created. To check the validity of the first designed codebook, the 2 coders individually tested it by examining the same sample of 100 tweets (10% of the overall data set). In the first attempt, intercoder agreement was low, so the 2 coders made several changes to the codebook after some discussion and deliberation. The first main change in the codebook was to reduce topics from 5 to 4, as we realized that 1 category focused on promotional and marketing messages belonged to the third topic. Following this, we renamed the second topic to include all types of calls to action and news related to lockdowns and protests against vaccinations. Finally, the descriptions of vaccine conspiracy theories were slightly revised to better represent what emerged from the Twitter data (Table 1). In the second attempt and with a modified codebook, an acceptable intercoder agreement was reached (Krippendorff α≥.774) [23].

**Table 1:** The codebook used in analyzing the Twitter data set.

Topic	Description
Infringing on civil liberties or perceived online censorship	Policies and government-imposed COVID-19 restrictions (eg, quarantine and travel restrictions) and online restrictions
Call for action or news on antilockdown and antivaccine protests	Joining or organizing a protest or rally or news about protests
Other	Minor issues or unrelated promotional messages
Vaccine conspiracy theories	The Deep State or the New World Order planning the pandemic for political gains Governments or pharmaceutical companies profiting from vaccines and intentionally risking general public health Attacking heath authorities or official medical news Mistrusting the efficacy of the vaccine or making false or exaggerated claims about vaccine risks and vaccine-related diseases Believing that unproven treatments (eg, eating healthy and exercising well) can prevent the virus

### Ethical Considerations

Ethics approval was not required for this study because it used publicly available data on social media. SFU's Ethics Board does not require ethics clearance for studies using data from public domains like Twitter.

## Results

### Topic Details

This study examined Arabic COVID-19 and vaccination disinformation discourse on Twitter. Through an analysis of antivaccination hashtags, we examined the main topics users discuss online to cover a gap in the literature regarding pandemic-related content in Arabic. To answer the study’s research question, the data analysis showed that infringement on civil liberties is the topic most engaged with, covering 41.1% (n=483) of the data and gathering 66,835 (41.1%) retweets. The second-most engaging topic is vaccine conspiracy theories, with 40.5% (n=476) of the data analyzed and a total of 62,336 (38.4%) retweets. The scope of vaccine conspiracy theories varied as mistrusting vaccine efficacy (263/476, 55.3%) attracted the most attention, followed by the government’s intentional chaos (110/476, 23.1%). Minor conversations focused on attacking health authorities (49/476, 10.3%), the Deep State (40/476, 8.4%), and unproven treatments (14/476, 2.9%). As for the third topic users discuss, tweets pushing a call for action cover 12.7% (n=149) of the tweets that were analyzed, garnering 19,733 (12.1%) retweets, while other unrelated content is the least present topic, with only 5.7% (n=67) of the tweets analyzed and a total of 13,531 (8.3%) retweets (see Table 2). To further understand the results, we delved into each topic to present examples explaining arguments against COVID-19 vaccines found in the data.

**Table 2:** Frequencies and percentages of the main topics discussed on Twitter.

Topic		Frequency, n/N (%)	Retweets, n/N (%)
Infringing on civil liberties		483/1175 (41.1)	66,835/162,435 (41.1)
Call for action		149/1175 (12.7)	19,733/162,435 (12.1)
Other		67/1175 (5.7)	13,531/162,435 (8.3)
**Vaccine conspiracies (n=476, 40.5%; 62,336, 38.4%, retweets)**			
	Deep State	40/476 (8.4)	4876/62,336 (7.8)
	Government’s intentional chaos	110/476 (23.1)	13,874/62,336 (22.3)
	Attacking health authorities	49/476 (10.3)	6248/62,336 (10.0)
	Mistrusting vaccine efficacy	263/476 (55.3)	35,505/62,336 (57.0)
	Unproven treatments	14/476 (2.9)	1833/62,336 (2.9)

#### Infringing on Civil Liberties

This is the most prevalent topic found in the data set: 483 (41.1%) of the tweets analyzed that had 66,835 (41.1%) retweets. Users here focus on topics concerning their civil liberties and freedoms, claiming that governments violated such rights following the pandemic. Particularly, users here denounce government efforts to force them to take any of the COVID-19 vaccines for different reasons. Some users claim their criticism of vaccine mandates stems from a suspicion of the vaccines’ safety and effectiveness, while others discuss their fundamental right as individuals to refuse vaccination regardless of whether vaccines are safe. For example, 1 user posted the following message, which was retweeted 680 times:

The vaccine prevents contagion and infection. What’s on the market currently does not do either. So don’t call it a vaccine, but call it “gene therapy,” which is closer to reality. Why force people to an experimental and unapproved gene therapy? Its long-term damage is unknown.

Another user, who self-identifies as a lawyer, said in a tweet retweeted 403 times:

For the millionth time and over and over again I personally don’t care who took the vaccine and who refuses to take it, my role is only limited to providing legal awareness for those who refuse to take the compulsory vaccine. And that no person or entity has the right to impose the vaccine on a person who doesn’t want it, according to the constitution, agreements and laws indicating that.

We can see here that both tweets refuse a mandatory vaccine but for different reasons. The first user is suspicious of its components, which intersects with our analysis of the fourth topic, which highlights vaccine conspiracy theories. The second user’s refusal comes from a legal principle rather than conspiracy theories or disinformation.

In a similar vein, users in this category argue against government restrictions that differentiate between the vaccinated and the unvaccinated. Examples of such rules include limiting travel and access to public spaces to vaccinated individuals only while forcing a workplace vaccine mandate. One of the most popular posts that was retweeted 576 times mentioned the following:

When a distinction is made between the vaccinated and the unvaccinated in employment as well as travel and shopping, it is considered indirect coercion and a type of humanitarian crime.

Here the user goes further in their condemnation by denouncing restrictions that they believe deny the unvaccinated their basic human and civil rights.

Looking at the origins of conspiracies that talk about civil liberties, there are 2 dominant narratives: In relation to the first narrative, the underlying theory is that each subsequent variant of COVID-19 was released by the government to prolong the pandemic and expand its powers [24]. By doing this, governments can allegedly restrict the unvaccinated and isolate them from the general population in a similar method to the concentration camps used in Nazi Germany.

The second narrative, focusing on the Great Reset, is similar to the first one in that governments are allegedly using the pandemic to maintain their powers. However, the Great Reset conspiracy takes this a step further and claims that the COVID-19 pandemic was started by a secret global government to cause a global economic collapse [25]. This will be achieved through the continual lockdowns, which will then allow the secret global government to implement a socialist world government for its own benefit. The Great Reset became a mainstream theory once a proposal from the 2020 Davos summit about a postpandemic reset went viral and became misconstrued as something sinister [25]. When taken together, these narratives have the potential to create doubt in the actions of their governments and to protect their own rights, as was seen in several examples from this study.

#### Vaccine Conspiracy Theories

The second-most recurring topic deals with tweets spreading vaccine conspiracy theories. This topic covers 40.5% (n=476) of the tweets analyzed, garnering 62,336 (38.4%) retweets. We noted the presence of different theories, from government and pharmaceutical actions and policies to intentionally create chaos and the existence of a network of actors exploiting the pandemic and vaccines to gain power and money, as well as attacks on health authorities, distrust in vaccines, and theories about alternatives to vaccinations. Although users tweeted about a range of different conspiracy theories, mistrusting the vaccine’s efficacy, false or exaggerated claims about vaccine risks and vaccine-related diseases, and governments and pharmaceutical companies profiting from vaccines and intentionally risking the general public health, appeared the most.

To further illustrate this finding, 1 of the most retweeted tweets with 607 retweets states:

By virtue of my studies as a medical assistant at Kuwait University and because of my specialization in the Department of Medical Laboratories and my work experience in the laboratory of viral diseases in Mubarak Hospital, I read the websites of vaccines manufacturers and discussed with a group of doctors and made my decision not to get vaccinated.

Although some social media posts express a general hesitancy in vaccines, tweets such as the previous one are more dangerous as they claim to be coming from an expert in the medical or pharmaceutical field. Other users went a step further, claiming that vaccines cause death to anyone getting them. In 1 example, retweeted 471 times, the user argues that a citizen died following a vaccine shot, tagging and attacking Kuwait’s former health minister Basel Al Sabah. The tweet says:

Dr. Basil @drbaselalsabah. A citizen took the vaccine and suffered a stroke and today she died. Do you still want to force people to be vaccinated! The tragedy is that there was a doctor mocking her condition and considered it a figment of her imagination.

In fact, COVID-19 vaccines can cause serious side effects in some people, but the reasoning is wrong because of overgeneralization.

Some tweets focus on attacking the Kuwaiti and Arab governments, criticizing how they handled the pandemic and accusing them of intentionally creating chaos. This tweet, which was retweeted 201 times, says:

A new heresy invented and approved by the government right away which is to prevent unvaccinated citizens from travelling. The confused government became an expert in creating crises and adding restrictions to citizens. I suggest they change their advisors because they sent the government into a vortex.

Again, the reasoning suggests that the government is intentionally trying to create chaos and social disruption.

When tracing the origins of vaccine-related conspiracy theories, the Center for Reality and Historical Studies, which is an online content hub that publishes disinformation about COVID-19, has been a reoccurring source of conspiracies. In 2021, the group uploaded a 27-minute video to Facebook, titled *Ask the Experts (COVID-19)*, which featured testimonials from 30 individuals who were credited as being doctors, health experts, and journalists. The key claims made in this video are that there was no pandemic so there could be no vaccine and that the industry skipped animal trials while producing the vaccine, so the harms are unknown; in addition, taking the vaccine will allegedly change your DNA [10]. The video subsequently went viral on Facebook, specifically among Arabic speakers.

A second prominent conspiracy theory about the vaccine is that pharmaceutical companies created COVID-19 to make the global populations ill and, in turn, increase their profits [18]. In the same vein, there are multiple conspiracy theories that believe that Bill Gates or the Gates Foundation created COVID-19 as a way to mass-vaccinate populations with a microchip. This in turn will allow Gates and the world government to track everyone at once [18]. For these conspiracy theories, Gates was singled out largely because of 2015 Ted Talk where he discussed the Ebola outbreak as a precursor of future pandemics, which individuals consider to be foreshadowing for the COVID-19 pandemic. These 2 examples, when taken along with statements from prominent individuals, such as the president of Lebanon and the leader of the Hezbollah [26], who express doubt over taking the vaccine, have created a strong foundation for the spreading of disinformation among users, as is shown in the results of this study.

#### Calls for Action

The third-most referenced topic includes tweets suggesting calls to action by users. These calls range from protesting to encouraging other users to take action about the vaccine mandate. Additionally, disseminating news about protests against COVID-19 measures happening worldwide falls under this topic. Overall, this topic covers 12.7% (n=149) of the tweets analyzed, garnering 19,733 (12.1%) retweets. Many tweets discuss a sit-in by individuals opposing the vaccine in Al-Erada Square, a public gathering square in Kuwait City in front of the Kuwaiti National Assembly Building. Historically, this public space has been used for protesting political causes [27], and after the pandemic, it became a popular place for protestors opposing the vaccine [28]. One of the most retweeted posts in this category, with 370 retweets, reads as follows:

I will show you the other side of the real sit-in, and unfortunately, last week most of the newspapers portrayed protestors badly. That’s why today I was in #Al-Erada_square and filmed with my personal camera a group of interviews that I will upload to my personal account which is not subject to any agenda like that of some newspapers.

Such tweets suggest the importance of this square as a spot for Kuwaitis opposing vaccines as well as the mainstream news media in Kuwait.

In another example, a user urged vaccinated individuals to speak about any health issues they faced after receiving the vaccine and suggested that several vaccinated individuals are suffering but afraid to talk. In a post retweeted 338 times, 1 Twitter user said:

To every person whose healthcare worsened following the vaccine but was told that this has nothing to do with the vaccine…don’t be afraid and speak loudly. A committee must be established to collect such cases and study them. People’s healthcare is not a game!!

Similar tweets encourage those against mandatory vaccinations to join protests and actively engage in different forms of peaceful civil disobedience. We also found 1 user promoting fake vaccine passports and offering their WhatsApp number for anyone interested in getting one as a form of action against state vaccination policies.

When tracing the logic behind calls for action, the narratives are not outwardly conspiratorial. These calls for action are rooted in a deep distrust of governments that is compounded by the increased restriction stemming from the pandemic. It is these feelings, along with the invocation of key phrases, that can lead to large-scale calls for action. An example of this process from outside the Middle East can be seen in the communications of 2 Austrian political parties. The People, Freedom, Fundamental Rights (MFG) Party has taken a staunch antivaccination stance and planned multiple protests using key words such as “dictatorship” and “apartheid” to rally large crowds [29]. In a similar vein, the Freedom Party (FPOe) in Austria has also planned large political events, building off a strong opposition to COVID-19 restriction and lockdowns. The party leader, Herbet Kickl, for example, has described Austria’s vaccination programs as a genetic experiment, further rallying individuals who may subscribe to similar conspiracy theories [29]. We argue here that the seeds of these ideas and their false claims are similar to what we have studied in Arabic social media posts around COVID-19 disinformation.

#### Co-opting of Hashtags

Finally, the last topic present in our analysis represents tweets from users aiming to take advantage of antivaccination hashtags to promote irrelevant commercial activities or even reach a wider audience. These tweets, which constituted 67 (5.7%) tweets from the total sample, were not related to the pandemic or vaccines in any sense but were merely marketing and promotional attempts or efforts to expand their online reach by using trending hashtags. For instance, 1 tweet promoted the service of a cleaning company, noting that it uses premium scented cleaning products to remove stains.

## Discussion

### Principal Findings

This study identified 3 prominent topics that were prevalent amongst Arabic speakers on Twitter. The first topic focused on civil liberties and governments’ alleged violations of freedom during the pandemic. Users who posted about this topic tended to express opposition toward vaccine mandates and increased restrictions for those who chose to remain unvaccinated. Those who posted about refusing the vaccine would often cite their legal rights or a lack of trust in the safety of the vaccine. There are also instances of individuals discussing a violation of their human rights due to vaccine mandates. When exploring the logic that underpins these narratives, there is a common theme that governments or pharmaceutical companies are using the pandemic as a way to expand their own powers or profits.

Moving to the category of conspiracy theories about the COVID-19 vaccine, we found that most common theories discussed are about the efficacy of the vaccine and the exaggerated risks that would come with taking it. With both of these narratives, credible individuals who self-identified as either doctors or authorities within the medical community discussed why they are not getting the vaccine and also shared some examples of individuals getting sick or dying after taking the vaccine. We also found instances where individuals accused their governments of intentionally mishandling the pandemic and creating restrictions for their own benefits. The logic used in these examples builds off the mistrust of governments that underpins the majority of the conspiracy theories that have been explored in this study. These theories have been further emboldened by examples of political officials publicly expressing their own hesitations in or rejection of taking the vaccine.

Finally, the third category that we identified is calls for action, in which we found that there are users encouraging others to participate in several different activities, such as rallies or protests. There is an underlying narrative of encouraging others to be brave in the face of intimidation and injustice when participating in these activities. Looking at the logic behind this type of disinformation, we found some similar narratives among far-right parties in the West that co-opted key phrases to build support for their own movements.

Looking at the results of our study, we find that our themes are similar to those identified in several other papers. Specifically, content that spreads rumors and conspiracy theories about governments and pharmaceutical companies has been identified in separate Twitter and Facebook studies, as well as in Egyptian social media pages [5,12]. Additionally, our category of infringing on civil liberties is similar to social media posts identified in another study that looked at social media posts in the Middle East and the North Africa region [11]. Although our study focused on tweets coming from what seems to be regular users, Grossman et al [11] examined posts disseminated by state-sponsored social media users.

Throughout our study, we noticed that the moderation policies toward COVID-19 misinformation in Arabic on Twitter are not strict, allowing people to post and maintain a variety of conspiracy theories.

### Study Limitations

This study was limited to the examination of a few Arabic hashtags on Twitter that imply doubt about the pandemic and its vaccines. Future research needs to focus on mobile apps that are popular in the Middle East, such as Telegram, Signal, and ClubHouse. To better understand the impact of COVID-19 disinformation on the Arab world, the experiences of many countries in the Arab world will need further exploration and reviewing platforms, such as Twitter, YouTube, Instagram, and WhatsApp. It will also be interesting to examine how COVID-19 misinformation in other languages, such as English, is translated, shared, and used by Arabic speakers. Finally, interviews with antivaxxers from the region are lacking, and more surveys based on cross-national comparative research in the Middle East are needed in order to obtain a clearer picture about the perceived views on the efficacy of vaccination and public health measures.

### Conclusion

Based on our findings, we were able to identify 3 prominent topics that were prevalent amongst Arabic speakers on Twitter. These categories focused on violations of civil liberties by governments, conspiracy theories about the vaccines, and calls for action. Our findings also highlight the need for more research to better understand the impact of COVID-19 disinformation on the Arab world.

## Appendix

Multimedia Appendix 1 Supplementary Figure 1.
