# Supplementary figures and images for: Investigation of COVID-19 Misinformation in Arabic on Twitter: Content Analysis
Source: JMIR Infodemiology. 2022 Jul 26;2(2):e37007. doi: 10.2196/37007 (PMC9327499; doi:10.2196/37007)

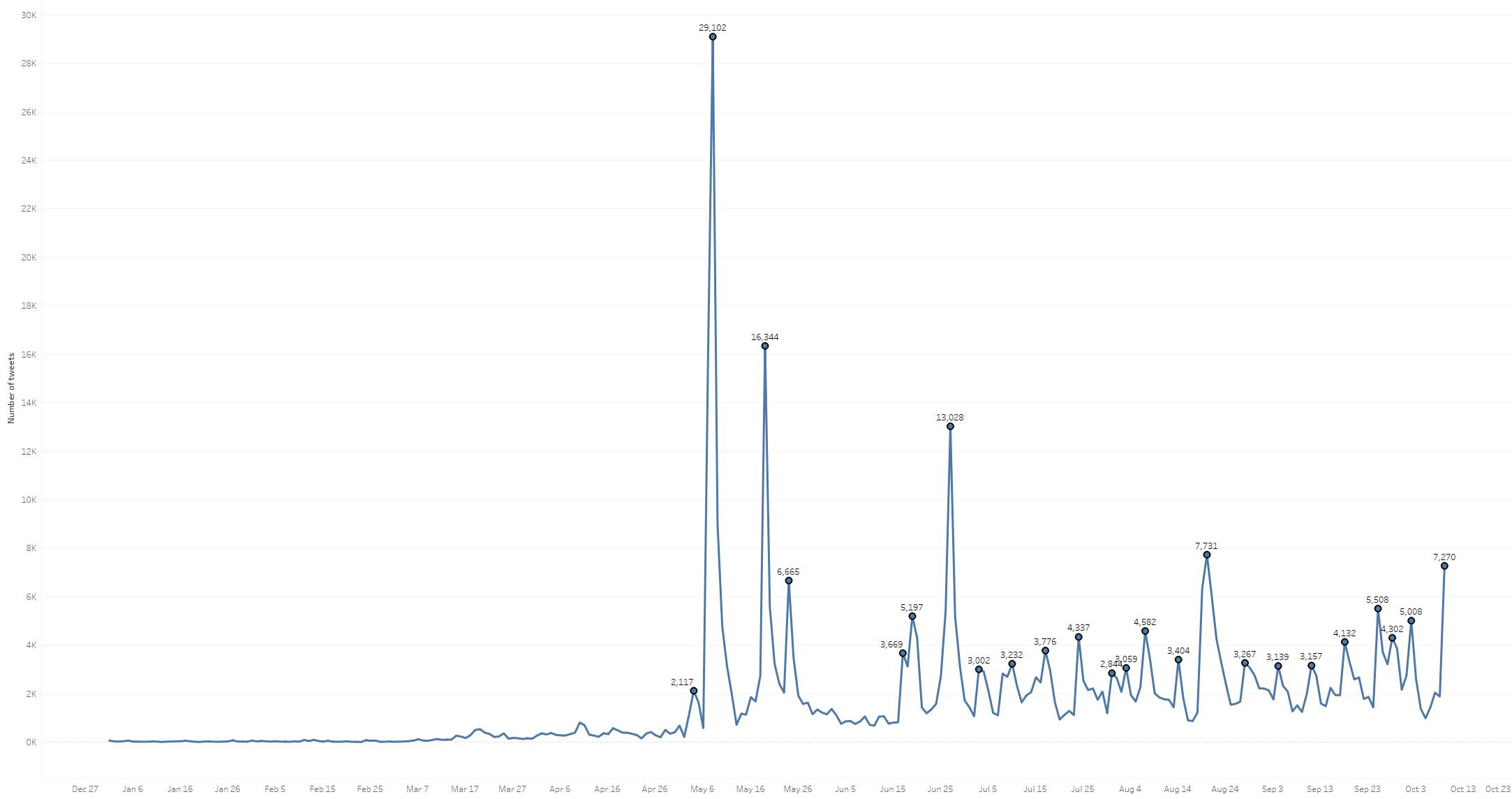

Supplement: Multimedia Appendix 1 [file infodemiology_v2i2e37007_app1.png]
